# Design of dual-effect molecules based on lysosome-targeting strategy: a mechanistic study on synergistic inhibition of ewing sarcoma and coronaviruses

**DOI:** 10.3389/fchem.2026.1851116

**Published:** 2026-06-19

**Authors:** Shimeng Zhou, Lu Xu, Wenlong Ge

**Affiliations:** 1 Cangzhou Integrated Traditional Chinese and Western Medicine Hospital, Hebei Key Laboratory of Integrated Traditional and Western Medicine in Osteoarthrosis Research (Preparing), Cangzhou, Hebei, China; 2 Cangzhou People’s Hospital, Cangzhou, Hebei, China

**Keywords:** antitumor, antiviral, dual activity, Hostorganelletargeting, Lysosometargeting

## Abstract

**Introduction:**

Addressing clinical challenges such as the toxicity of conventional chemotherapy and viral drug resistance, this study proposes and validates an innovative “host organelle-targeting” strategy. By disrupting the function of host lysosomes—an organelle commonly exploited by both tumors and viruses—we aim to develop novel therapeutic agents with dual antitumor and antiviral activity.

**Methods:**

We rationally designed and synthesized the target compound 3-chloro-4-(4-(diethylamino)butoxy)benzaldehyde, whose core design relies on the pH-responsive property of the 4-(diethylamino)butoxy side chain to enable specific enrichment within the acidic lumen of lysosomes. *In vitro* activity was evaluated against Ewing sarcoma cells (TC32), prostate cancer bone metastasis model cells, porcine epidemic diarrhea virus (PEDV), and transmissible gastroenteritis virus (TGEV). Mechanistic investigations assessed lysosomal pH, autophagic flux, endoplasmic reticulum stress-related gene expression, p53 signaling pathway, cell cycle, and apoptosis.

**Results:**

The compound potently inhibited the proliferation of TC32 cells (IC_50_ = 25 μM) and prostate cancer bone metastasis cells, and significantly suppressed the replication of PEDV and TGEV. Mechanistic studies revealed that the compound neutralizes lysosomal pH, impairs acidification, blocks autophagic flux, upregulates endoplasmic reticulum stress-related gene expression, activates the p53 signaling pathway, and inhibits the cell cycle, ultimately leading to apoptosis.

**Conclusion:**

This study reports a chemical entity with well-defined dual antitumor and antiviral activities and provides mechanistic evidence supporting the feasibility of the “host-lysosome targeting” strategy. It thus offers an important proof-of-concept and a research framework for developing broad-spectrum host-directed therapies.

## 1 Introduction

In the context of interdisciplinary integration in biomedical sciences, orthopedic oncology is continuously incorporating new scientific perspectives to address persistent clinical challenges (Siegel et al., 2013; Watts et al., 2017). Ewing sarcoma, a highly aggressive bone tumor predominantly affecting children and adolescents, is molecularly characterized by fusion gene drivers such as EWSR1-FLI1 (Gartrell and Rodriguez-Galindo, 2021; Kim and Park, 2016). The current multimodal treatment, primarily based on intensive chemotherapy, surgery, and radiotherapy, shows limited efficacy against metastatic or recurrent disease. This therapeutic impasse largely stems from the broad toxicity of conventional chemotherapy, coupled with tumor heterogeneity and acquired resistance (De Las Rivas et al., 2021; Pan et al., 2021). Consequently, there is an urgent need for agents with novel mechanisms of action. Ideally, such agents should circumvent existing resistance mechanisms or target more fundamental, common cellular processes essential for tumor cell survival (Buck et al., 2021; Daniyal et al., 2021). In parallel, the field of virology faces analogous challenges. Coronaviruses, exemplified by the porcine epidemic diarrhea virus (PEDV), rely heavily on the host cell’s endocytic-lysosomal system for entry and replication (Turlewicz-Podbielska and Pomorska-Mól, 2021; Wang et al., 2016). This reveals a profound biological parallel: both rapidly proliferating tumor cells and efficiently replicating viruses place exceptional demands on, and actively remodel, fundamental host cell resources and regulatory networks, such as lysosomal function.

Building on this, shifting the therapeutic strategy from the traditional “direct targeting of pathogens or oncoproteins” to “targeting host factors that are commonly exploited by both tumors and viruses” has emerged as a promising direction (Ghimire et al., 2018; Zhou and Zhang, 2016). Among these, the lysosome—a cellular “digestion and recycling center” and signaling hub—may become vulnerable under the intense pressure imposed by rapid proliferation or viral hijacking, thereby representing a novel and potentially broad-spectrum intervention point (Perera and Zoncu, 2016; Yang and Wang, 2021). This leads to a core scientific hypothesis: Can a small molecule be designed to specifically disrupt the function of this shared host organelle, the lysosome, thereby exerting a synergistic dual inhibitory effect on tumor cell proliferation and viral replication? Such a “host-directed” dual-activity molecule not only holds promise for overcoming the limitations of conventional therapies but may also reveal a fundamental cell biological principle. To test this hypothesis, this study proposes and implements an innovative “host organelle targeting” drug design strategy. We selected 4-hydroxybenzaldehyde—a structurally simple and readily derivatizable scaffold—as the parent core and performed rational structural optimization by introducing key pharmacophores: a chlorine atom was introduced onto the benzene ring to modulate physicochemical properties, while a 4-(diethylamino)butoxy side chain containing a tertiary amine group was designed and incorporated. This side chain confers a unique “lysosome-targeting” capability: the tertiary amine group remains membrane-permeable at physiological pH but becomes protonated and trapped within the acidic lumen of the lysosome, where it accumulates and disrupts lysosomal function by neutralizing the acidic environment (Khan et al., 2018; Stewart et al., 2025).

We successfully synthesized the target compound 3-chloro-4-(4-(diethylamino)butoxy)benzaldehyde, aiming to systematically evaluate whether it could achieve the predicted “dual activity.” For the antitumor assessment, we selected Ewing sarcoma (TC32, A673, RD-ES) and prostate cancer bone metastasis model (PC3, DU145) cell lines to evaluate its anti-proliferative activity. For the antiviral assessment, we employed PEDV and transmissible gastroenteritis virus (TGEV) infection models to measure its efficacy in inhibiting viral replication. Furthermore, by integrating transcriptomics, cellular functional assays, and organelle-level detection, we deeply investigated the common molecular mechanism underlying its simultaneous antitumor and antiviral effects across multiple dimensions, including lysosomal acidification status, autophagic flux, endoplasmic reticulum stress, the p53 signaling pathway, and the cell cycle. This study not only reports a novel chemical entity with dual activity but also aims, through mechanism-driven exploration, to provide a proof-of-concept and a new framework for developing broad-spectrum therapeutic strategies based on “host organelle targeting.”

## 2 Results and discussion

### 2.1 Chemical synthesis

**FIGURE 1:**
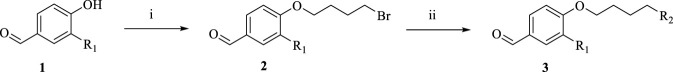
Design and synthesis of 4-(diethylamino)butoxy derivative. Conditions and reagents: i) acetone, K_2_CO_3,_ 50 °C, yield 70%–82%; ii) acetone, different aldehyde groups, 50 °C, yield 61%–80%.

Based on a “host organelle-targeting” strategy, we successfully designed and synthesized the compound 3-chloro-4-(4-(diethylamino)butoxy)benzaldehyde. This molecule utilizes 4-hydroxybenzaldehyde-readily derivatizable and possessing favorable membrane permeability—as its core scaffold. A chlorine atom was introduced at the para position of the benzene ring to modulate lipophilicity and potentially engage in halogen-bonding interactions. The central design feature is the incorporation of a 4-(diethylamino)butoxy side chain. The tertiary amine group within this chain remains uncharged and membrane-permeable at physiological pH but becomes protonated and positively charged in acidic compartments such as lysosomes, leading to its specific “trapping” and accumulation within these organelles, thereby achieving pH gradient-dependent lysosomal targeting (Kroemer and Galluzzi, 2017; Liu et al., 2021). We hypothesized that the high intra-lysosomal concentration of this compound might disrupt lysosomal homeostasis *via* mechanisms such as compromising membrane integrity, neutralizing the acidic pH, or interfering with autophagic flux. This dual perturbation could simultaneously affect the survival of tumor cells reliant on lysosomal function and the lifecycle of viruses dependent on lysosomes for entry. The specific synthetic route is illustrated in Figure 1, starting from 4-hydroxybenzaldehyde, followed by the introduction of a linear carbon chain at the hydroxyl group, and subsequent reaction with the appropriate amine derivative to yield the final product **3**.

### 2.2 Antiproliferative screening of compounds

Guided by the innovative strategy of “targeting host organelles,” we first systematically evaluated the antiproliferative activity of novel compounds, designed based on the 4-hydroxybenzaldehyde scaffold, against two representative tumor models (Fu and Wang, 2023). Ewing sarcoma cell lines (TC32, A673, RD-ES) and prostate cancer bone metastasis model cell lines (PC3, DU145) were selected for assessment, with the parent core molecule and the weakly active 4-Diethylaminobenzaldehyde (**DEAB**) serving as references. Initial screening results demonstrated that the modified compounds exhibited significantly superior inhibitory activity compared to the reference agents (Table 1). Among them, the lead compound **3b** showed the most potent activity against the Ewing sarcoma TC32 cells (IC_50_ = 25 µM), representing an over 8-fold increase in potency compared to the parent core. Compound **3b** also displayed strong and specific inhibition against prostate cancer PC3 cells (IC_50_ = 38 µM). Based on these findings, subsequent investigations were focused on the lead compound **3b** to delve deeper into its antitumor mechanism.

**TABLE 1:** Antiproliferative effect of DEAB analogues on various cancer cell lines.

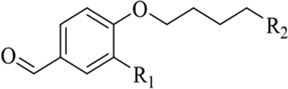							
Compounds	IC_50_ (μM)						
	R1	R2	TC32	A673	RD-ES	PC3	DU145
**DEAB**	—	—	>200	>200	>200	>200	>200
**3a**	H	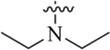	89 ± 4	70 ± 6	80 ± 5	76 ± 5	60 ± 3
**3b**	Cl	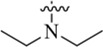	25 ± 3	28 ± 5	40 ± 5	38 ± 6	48 ± 4
**3c**	H	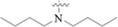	101 ± 6	28 ± 5	40 ± 5	38 ± 6	51 ± 4
**3d**	Cl	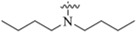	70 ± 6	37 ± 4	51 ± 6	50 ± 7	55 ± 3

Data are presented as mean ± SD from three independent experiments (n = 3).

### 2.3 Compound 3b inhibits TC32 cell proliferation and induces apoptosis

**FIGURE 2:**
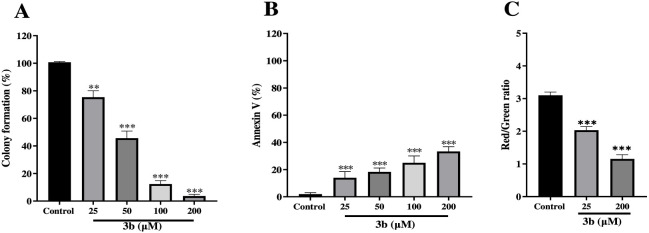
**(A)** Statistical graph of the relative colony formation rate in TC32 cells; **(B)** Significant induction of apoptosis by compound **3b** in TC32 cells; **(C)** JC-1 analysis of mitochondrial membrane potential. Data are presented as mean ± SEM from three independent experiments (n = 3). Compared with the Control group, *P < 0.05, **P < 0.01, and ***P < 0.001.

A colony formation assay was performed to evaluate the effect of compound **3b** on TC32 cells at concentrations of 25, 50, 100, and 200 μM. As shown in Figure 2A, treatment with 3b at this concentration gradient significantly reduced the number of cell colonies in a dose-dependent manner. Colony formation was almost completely inhibited at the concentration of 200 μM. Furthermore, analysis of apoptosis by flow cytometry indicated that treatment with 200 μM **3b** induced an apoptotic rate of 33% in TC32 cells, whereas the proportion of apoptotic cells in the DMSO control group was very low (less than 4%) (Figure 2B). The detailed distribution of live, early apoptotic, late apoptotic, and necrotic cells is provided in Supplementary Table S1. To further validate the apoptosis-inducing effect, we measured the mitochondrial membrane potential using the JC-1 probe. As shown in Figure 2C, compound **3b** decreased the red/green fluorescence ratio in a concentration-dependent manner. The ratio decreased from 3.1 in the control group to 2.0 in the 25 μM group, and further to 1.2 in the 200 μM group. These results indicate that compound **3b** causes a loss of mitochondrial membrane potential, an early event in the apoptotic cascade. Collectively, these results demonstrate that compound **3b** significantly inhibits the proliferation of TC32 cells.

### 2.4 Antitumor mechanism of the compound 3b

**FIGURE 3:**
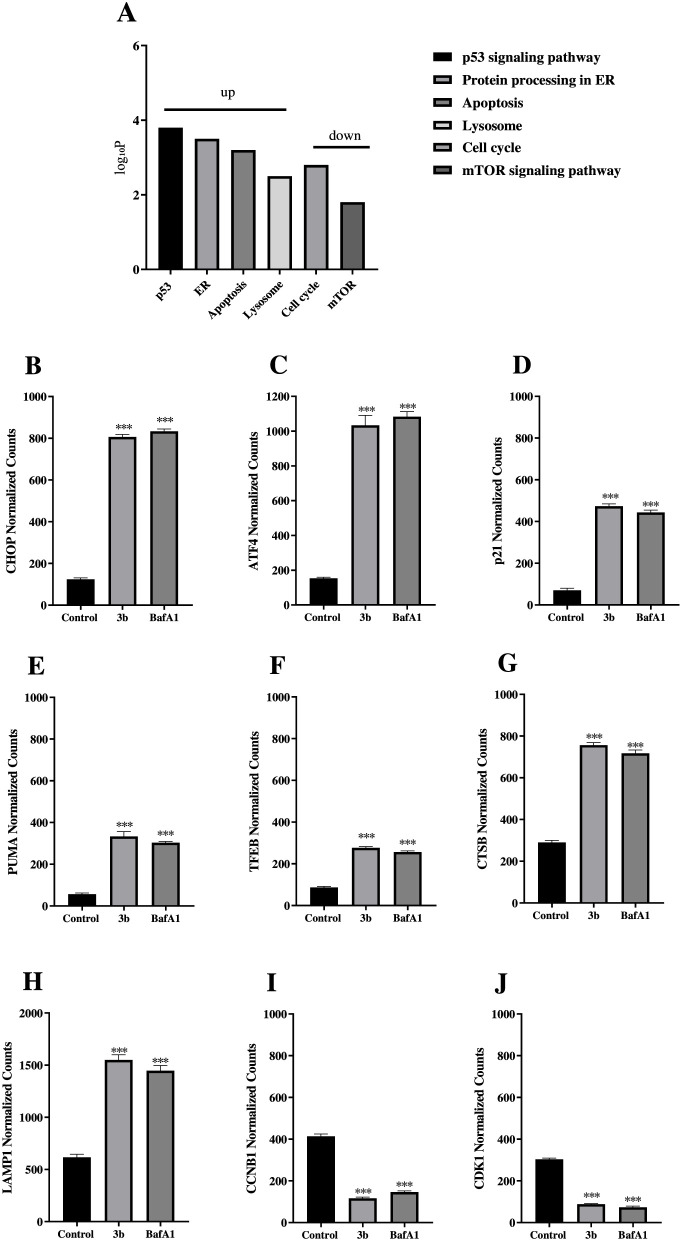
**(A)** Bar graph showing the significance of pathway enrichment. Key differentially expressed genes were validated according to functional modules. **(B,C)** Representative gene expression levels of the endoplasmic reticulum stress response, **(D,E)** the p53 signaling pathway, **(F–H)** lysosome biogenesis, and **(I,J)** cell cycle progression are shown. Data are presented as mean ± SEM from three independent experiments (n = 3). *p < 0.05, **p < 0.01, ***p < 0.001 compared with the Control group.

KEGG pathway enrichment analysis revealed that the most significantly activated pathways upon compound **3b** treatment (ranked by FDR) included the p53 signaling pathway, endoplasmic reticulum protein processing, and apoptosis (FDR <0.001) (Figure 3A). Consistently, genes associated with the lysosomal pathway were also significantly upregulated (FDR <0.01). In contrast, the cell cycle pathway was markedly suppressed. To validate the RNA-seq data and further delineate the action profile of compound **3b**, we performed quantitative analysis of hallmark genes in these key pathways (Figures 3B,C). Within the ER stress pathway, compound **3b** treatment elevated the expression levels of CHOP and ATF4 by 6.7-fold (p < 0.001), an induction comparable to that elicited by BafA1 treatment (ns). Similarly, in the p53 signaling pathway, its downstream effectors p21 and PUMA were robustly upregulated by approximately 6-fold (p < 0.001) (Figures 3D,E). Notably, the expression of TFEB—the master transcriptional regulator of lysosomal biogenesis—and its target genes CTSB and LAMP1 exhibited a consistent and significant co-upregulation (2.5- to 3-fold, p < 0.001) (Figures 3F–H), indicating the activation of a transcriptional reprogramming likely aimed at compensating for lysosomal functional impairment. In sharp contrast, the expression of key drivers of cell cycle progression, CCNB1 and CDK1, was significantly suppressed to about 30% of the control level (p < 0.001) (Figures 3I,J). It should be noted that these findings are based on transcriptional analysis; protein-level validation of ER stress markers (e.g., ATF4 and CHOP) was not performed in this study. For all genes examined, no statistically significant differences were observed between the compound **3b**- and BafA1-treated groups, confirming at the transcriptional level that both treatments triggered highly similar, widespread cellular stress and growth arrest responses by disrupting lysosomal function.

### 2.5 Initial screening of antiviral activity

To verify the potential broad-spectrum antiviral activity of the compounds designed based on the host-lysosome-targeting strategy, we evaluated their efficacy against porcine epidemic diarrhea virus (PEDV) and transmissible gastroenteritis virus (TGEV) (Yang et al., 2017). As shown in Table 2, the compound **3b** exhibited significant inhibitory activity against both PEDV and TGEV at non-cytotoxic concentrations, effectively reducing viral replication markers. Specifically, compound **3b** exhibited a CC_50_ of 301 μM in normal Vero cells, which was approximately 12-fold higher than its IC_50_ in TC32 cancer cells (25 μM), indicating a favorable therapeutic window with preferential cytotoxicity toward tumor cells. The half-maximal effective concentration (EC_50_) fell within a range similar to that observed for its antitumor activity. These parallel activity data indicate that the biological effect of the compound **3b** is not confined to tumor cells but extends to viral infections that also critically depend on lysosomal function. Therefore, the results in this section not only independently confirm the definite antiviral efficacy of the compound, but more importantly, they effectively link the “lysosomal perturbation” mechanism previously established in tumor models to a new pathological context—viral infection. This strongly supports our core hypothesis that by targeting the host lysosome as a common cellular hub, **3b** can simultaneously exert dual antitumor and antiviral effects, thereby providing key experimental evidence for the development of a broad-spectrum, host-directed therapeutic strategy.

**TABLE 2:** Antiviral activity of DEAB analogues against PEDV and TGEV.

Compounds	PEDV EC_50_ ^a^ (μM)	PEDV CC_50_ ^b^ (μM)	PEDV SI^c^	TGEV EC_50_ (μM)	TGEV CC_50_ (μM)	TGEV SI
DEAB	>200	>200	—	>200	>200	—
3a	72	603	8.4	>200	>200	—
3b	5	301	60.2	155	685	4.4
3c	>200	>200	—	>200	>200	—
3d	2	10.8	5.4	187	895	4.8

Data are presented as mean ± SD, from three independent experiments (n = 3).

^a^ EC_50_ = 50% effective concentration (concentration at which 50% inhibition of TGEV, or PEDV, are observed).

^b^ CC_50_ = 50% cytostatic/cytotoxic concentration (concentration at which 50% adverse effect is observed on the host cell).

^c^ SI: selectivity index (CC_50_/EC_50_).

### 2.6 3b inhibits late-stage viral gene transcription

**FIGURE 4:**
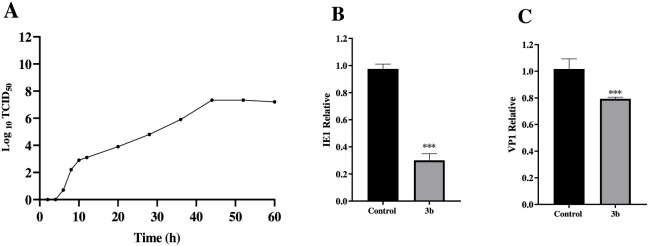
**(A)** One-step growth curve of PEDV in the presence of compound **3b**. **(B)** Relative mRNA expression levels of the viral early gene (*IE1*), measured by qRT-PCR at 12 h post-infection in cells pretreated with compound **3b** or DMSO and infected with PEDV. **(C)** Relative mRNA expression levels of the viral late gene (*VP1*), measured by qRT-PCR at 24 h post-infection. Data are presented as mean ± SEM from three independent experiments (n = 3). Compared with control, *p < 0.05, **p < 0.01, ***p < 0.001.

To assess the effect of compound **3b** on PEDV replication, we measured the time-course changes in viral titer in infected cells treated with the compound. Vero cells were pretreated with compound **3b** or DMSO control and then infected with PEDV at the same multiplicity of infection (MOI). Virus-containing supernatants were collected at various time points post-infection (0, 6, 12, 24, 36, 44, and 52 h), and viral titers were determined by the TCID_50_ assay. The one-step growth curve (Figure 4A) showed that PEDV could still complete a full replication cycle in the presence of the compound. After a latent period of approximately 6 h post-infection, the viral titer rose sharply, entering the exponential phase and peaking at 44 h (7.33 Log_10_ TCID_50_), followed by a plateau phase. Subsequent qPCR results (Figures 4B,C) revealed that compound **3b** specifically and strongly suppressed the transcription of the late viral gene (*VP1*), while exerting a lesser effect on the early gene (*IE1*). We therefore conclude that compound **3b** disrupts the host cellular environment required for viral replication, particularly during the late stage.

### 2.7 Targeting lysosomes

**FIGURE 5:**
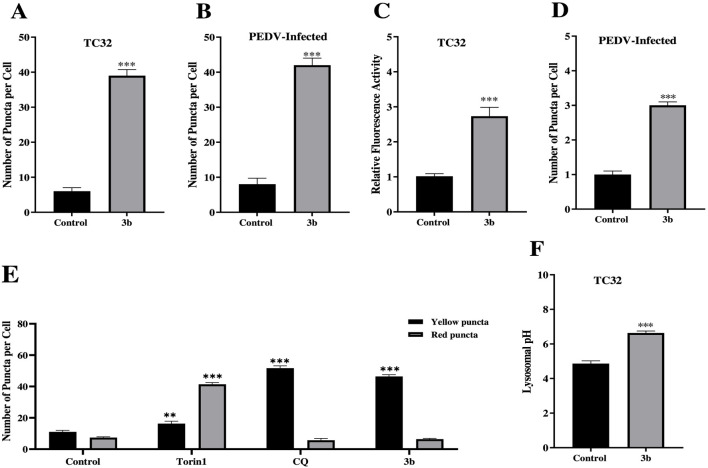
**(A,B)** Analysis of Galectin-3 immunofluorescent puncta. **(A)** TC32 cells and **(B)** PEDV-infected Vero cells were treated with DMSO or compound 3b (50 μM, 24 h), followed by immunofluorescence staining for Galectin-3. **(C,D)** Measurement of cytosolic cathepsin B/D activity. **(C)** TC32 cells and **(D)** PEDV-infected Vero cells were treated under the same conditions as in **(A,B)**. Cytosolic protein fractions were isolated, and their enzymatic activity was determined using a cathepsin B/D-specific fluorescent substrate. **(E)** Analysis of autophagic flux using the mRFP-GFP-LC3 tandem fluorescent probe. The average numbers of yellow and red puncta per cell were quantified. **(F)** Quantitative analysis of intralysosomal pH. Lysosomal pH was quantified using the pH-sensitive fluorescent probe LysoSensor Yellow/Blue. Data are presented as fold change relative to the DMSO control group. Data are presented as mean ± SEM from three independent experiments (n = 3). Compared with control, *p < 0.05, **p < 0.01, ***p < 0.001.

To quantitatively assess the effect of the compound **3b** on the autophagy process, we measured both the number of puncta of the autolysosome marker Galectin-3 and the activity of key lysosomal enzymes. First, quantitative analysis of Galectin-3 immunofluorescence (Figures 5A,B) showed that compound **3b** treatment significantly increased the average number of Galectin-3 puncta per cell compared with the respective solvent control (DMSO) in both TC32 cells and PEDV-infected Vero cells (from ∼6 to ∼39 puncta/cell in TC32 cells, and from ∼8 to ∼42 puncta/cell in PEDV-infected Vero cells). This indicates that the compound **3b** effectively induced the accumulation of Galectin-3-positive structures. To further investigate the functional status of lysosomes downstream of autophagy, we detected the activity of cytosolic cathepsin B/D. As shown in Figures 5C,D, compound **3b** treatment also markedly enhanced the relative fluorescent activity of cathepsins in both cell models. When the activity of each control group was set as 1, compound **3b** treatment increased the enzymatic activity to 2.7-fold and 3-fold, respectively (*p < 0.001). Together, these quantitative data demonstrate that the compound **3b** not only promotes the formation of autolysosome-related structures but is also accompanied by enhanced lysosomal degradative activity. To clarify the effect of the compound **3b** on autophagic flux, we performed quantitative analysis of cells labeled with the mRFP-GFP-LC3 probe. As shown in Figure 5E, compared with the DMSO control, treatment with the autophagy inducer Torin1 dramatically increased the number of red puncta (41 vs. 7), while yellow puncta showed only a moderate increase, consistent with successful activation and completion of autophagic flux. In contrast, treatment with the lysosomal inhibitor chloroquine (CQ) exhibited a distinctly different pattern: yellow puncta accumulated sharply (52 vs. 11), whereas red puncta were suppressed. Importantly, our compound **3b** treatment completely reproduced this characteristic pattern of CQ: it similarly induced a pronounced accumulation of yellow puncta (46 vs. 11) and kept the number of red puncta at a low level, showing no statistical difference from the CQ group and being significantly lower than the Torin1-treated group. These quantitative data indicate that the compound **3b** does not promote smooth autophagic flux but, similar to CQ, causes accumulation of autophagosomes (yellow puncta) and a reduction in autolysosome (red puncta) formation, strongly suggesting that autophagic flux is blocked at the lysosomal degradation stage. MThe acidic environment of lysosomes is a prerequisite for their degradative function. To directly investigate whether the compound **3b** affects lysosomal acidification, we employed the pH-sensitive fluorescent probe LysoSensor for detection. As shown in Figure 5F, in DMSO-treated control cells, lysosomes displayed bright punctate fluorescent signals, indicating that their lumen maintained strong acidity. Quantitative analysis confirmed that compound **3b** treatment significantly raised the average lysosomal pH from 4.9 in the control group to 6.6. This result directly proves that the compound **3b** effectively disrupts the acidic environment of lysosomes. Together with the previous findings—that compound **3b** treatment elevated cathepsin activity and that autophagic flux was blocked at the lysosomal stage (mRFP-GFP-LC3 assay)—we conclude that the compound **3b** neutralizes the intra-lysosomal acidity, impairing its essential biochemical and degradative functions, thereby preventing the completion of autophagic flux.

### 2.8 Subcellular fractionation directly validates lysosomal targeting

**FIGURE 6:**
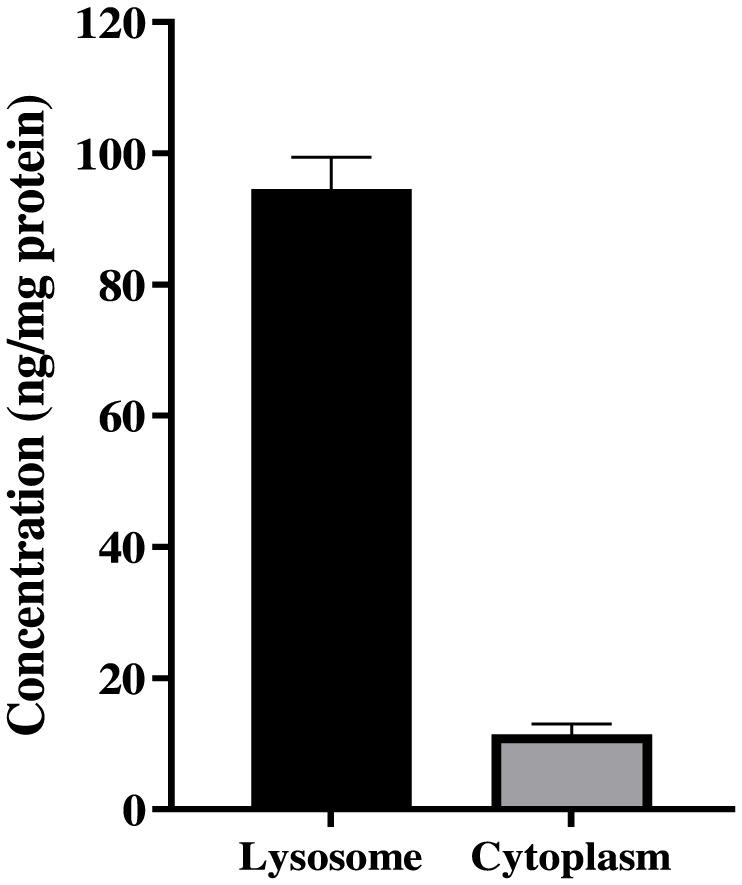
Distribution of compound **3b** in lysosomal and cytoplasmic fractions. Data are presented as mean ± SEM from three independent experiments (n = 3).

To directly verify the lysosomal localization of compound **3b**, organelle fractions were isolated by Percoll density gradient centrifugation and analyzed by LC-MS/MS. The results showed that the concentration of **3b** in the lysosomal fraction was 8.3-fold higher than that in the cytoplasmic fraction (Figure 6). These findings directly demonstrate that **3b** undergoes specific lysosomal accumulation *via* the ion-trapping mechanism.

## 3 Conclusion and discussion

This study successfully translated the therapeutic concept of “host organelle targeting” into a defined chemical entity with dual biological activities. A systematic structure-activity relationship (SAR) analysis revealed that each structural component contributes distinctively to the overall pharmacological profile. The tertiary amine-containing 4-(diethylamino)butoxy side chain is essential for lysosomal tropism, enabling pH-gradient-dependent accumulation within acidic organelles. The chlorine atom on the benzene ring modulates lipophilicity and may facilitate halogen-bonding interactions with putative targets, but its substitution pattern critically influences the therapeutic window: while compound 3d exhibited the most potent anti-PEDV activity (EC_50_ = 2 µM), its high cytotoxicity (CC_50_ = 10.8 µM) yielded a poor selectivity index (SI = 5.4); in contrast, compound **3b** achieved an optimal balance between efficacy and safety (EC_50_ = 5 μM, CC_50_ = 301 μM, SI = 60.2), suggesting that moderate lysosomal accumulation with reduced off-target toxicity is preferable. The aldehyde moiety may also contribute as a weak electrophile, potentially engaging in reversible covalent interactions with lysosomal proteins.

By virtue of its rationally designed 4-(diethylamino)butoxy side chain, 3-chloro-4-(4-(diethylamino)butoxy)benzaldehyde achieves pH-gradient-dependent targeting and accumulation in lysosomes. Its intra-lysosomal sequestration, which neutralizes the acidic luminal environment, acts as the initial trigger for the subsequent cascade of cellular events. Ultimately, this action disrupts autophagic flux, elicits an integrated stress response, and induces cell-cycle arrest, thereby synergistically suppressing both tumor cell proliferation and viral replication—processes that are highly dependent on functional lysosomes. Notably, we observed shared early organelle-dysfunction phenotypes and similar downstream signaling activation in two distinct pathological models (tumor and viral infection), providing strong support for the inherent logic of targeting common host vulnerabilities. Therefore, this work not only identifies a promising lead compound, but more importantly, offers multidimensional proof-of-concept—from chemical design to biological mechanism and pharmacological outcome—for a novel broad-spectrum therapeutic paradigm centered on perturbing host cellular homeostasis. From a pharmaceutical perspective, we acknowledge that the tertiary amine functionality present in compound **3d** may pose potential risks related to the formation of genotoxic impurities such as nitrosamines during synthesis or storage. In the current study, HPLC purity analysis (>95%) did not reveal any significant peaks corresponding to such impurities. For future development, comprehensive impurity profiling and risk assessment in accordance with ICH M7 guidelines will be essential to ensure safety.

## 4 Experimental section

### 4.1 Chemical synthesis

Under a nitrogen atmosphere, compound **1** (1 mmol) was dissolved in 10 mL of anhydrous acetone containing potassium carbonate (3 mmol). 1,4-Dibromobutane (3 mmol) was then added, and the mixture was heated under reflux for 6 h. After the reaction was confirmed to be complete by TLC monitoring, the solvent was removed under reduced pressure. The crude product was purified by silica gel column chromatography to afford compound **2**. Subsequently, compound 2 (1 mmol) was dissolved in 10 mL of anhydrous acetone. Diethylamine (0.5 mL) was added, and the reaction was stirred at 50 °C for 6 h. Upon completion, the mixture was washed twice with brine. The organic layer was dried, and the solvent was evaporated under reduced pressure. Purification by column chromatography yielded compound **3a** as a pale-yellow solid (145 mg, yield: 61%). Compounds **3b**–**3d** were isolated using a similar procedure. The ^1^H and ^13^C NMR spectra and data of the compound are provided in the **Supporting Information**.

### 4.2 Screening for antiproliferative activity of compounds

The antiproliferative activity of the compounds was evaluated using the CCK-8 assay. Cells of TC32, A673, RD-ES, PC3, and DU145 lines in the logarithmic growth phase were seeded into 96-well plates. After 24 h of incubation to allow cell adherence, the culture medium was replaced with fresh medium containing a series of concentrations of the test compounds (novel 4-hydroxybenzaldehyde derivatives, the parent core molecule, and DEAB), with solvent controls included. The cells were then cultured for an additional 48 h. Following this treatment, CCK-8 solution was added to each well. After an appropriate incubation period, the absorbance of each well was measured at 450 nm using a microplate reader. Cell viability rates were calculated based on the absorbance readings, and the half-maximal inhibitory concentration (IC_50_) values for each compound were determined by fitting the data to a nonlinear regression model. Based on the preliminary screening results, compound **3b** was selected for further investigation into its mechanism of action (Amrutha Nisthul et al., 2021; Chripkova et al., 2016).

### 4.3 Colony formation assay

TC32 cells in the logarithmic growth phase were seeded into 6-well plates. Following cell attachment, the cultures were treated with complete medium containing 25, 50, 100, and 200 μM of compound **3b**, respectively, with a DMSO solvent control group established in parallel. The compound-containing medium was replenished every 3 d. The cells were cultured for 10–14 d until distinct colonies formed in the control group. Subsequently, the culture was terminated. The medium was aspirated, and the cells were washed with PBS, fixed with 4% paraformaldehyde, and stained with 0.1% crystal violet. After air-drying, the plates were photographed, and the number of colonies in each group was counted (Liu et al., 2015; Rong et al., 2008).

### 4.4 Detection of cell apoptosis

TC32 cells were seeded into 6-well plates and cultured until reaching approximately 70% confluency. The culture medium was then replaced with fresh medium containing 200 μM of compound **3b**; a parallel control group was treated with an equivalent concentration of DMSO. After 24 h of treatment, both the culture supernatant and the adherent cells were collected. The cells were subsequently stained using an Annexin V-FITC/PI apoptosis detection kit, following the manufacturer’s instructions, and incubated at room temperature in the dark for 15 min. Apoptotic cell populations, including early and late apoptotic cells, were then analyzed by flow cytometry (Liu et al., 2015; Rong et al., 2008).

### 4.5 Bioinformatics analysis

Total RNA was extracted from TC32 cells treated with compound **3b** and control cells. Strand-specific libraries were constructed and subjected to high-throughput sequencing on an Illumina platform (RNA-seq). The raw sequencing reads were aligned to the reference genome using Hisat2, and gene expression was quantified with featureCounts. Differentially expressed genes (DEGs) were identified with the thresholds of |fold change| > 2 and adjusted *p*-value (FDR) < 0.05. The list of DEGs was then subjected to KEGG pathway enrichment analysis using tools such as KOBAS. The significance of enrichment was calculated by the hypergeometric test, and the *p*-values were adjusted for multiple testing using the Benjamini–Hochberg method to control the false discovery rate (FDR). Pathways with an FDR < 0.05 were considered significantly enriched (Huang et al., 2018; Zolfaghari Emameh et al., 2022).

### 4.6 RT-qPCR validation

To validate the reliability of the RNA-seq data, quantitative analysis was performed on marker genes from key pathways. TC32 cells from the control group, compound **3b**-treated group, and the positive control (BafA1)-treated group were collected. Total RNA was extracted using TRIzol reagent, and its concentration and purity were measured. An equal amount of RNA was reverse-transcribed into first-strand cDNA using a reverse transcription kit. Specific primers were designed with software such as Primer Premier 5.0 and synthesized commercially. GAPDH or β-actin was used as the internal reference gene. Amplification was carried out with SYBR Green Master Mix on a real-time PCR instrument under the following cycling conditions: 95 °C for 5 min; followed by 40 cycles of 95 °C for 10 s and 60 °C for 30 s. Each sample was run in triplicate, and a no-template control was included in each run. The relative expression levels of the target genes were calculated using the 2^−ΔΔCT^ method. The experiment was independently repeated three times (Huang et al., 2018; Zolfaghari Emameh et al., 2022).

### 4.7 Antiviral activity screening assay

Cytotoxicity Assay of Compounds (CC_50_): To evaluate compound cytotoxicity, host cells (Vero E6) were seeded into 96-well plates. After adherence, the culture medium was replaced with medium containing a series of concentrations of the test compounds, and the cells were incubated for 48–72 h. Cell viability was measured using the CCK-8 assay. The half-maximal cytotoxic concentration (CC_50_) was calculated by fitting the dose-response curve.

Antiviral Activity Assay of Compounds (EC_50_): To assess the inhibitory activity of compounds on viral replication, porcine epidemic diarrhea virus (PEDV) or transmissible gastroenteritis virus (TGEV) was simultaneously added with a series of compound concentrations to a monolayer of cells in 96-well plates. After co-cultivation for a specified period, the level of viral replication was evaluated by observing the cytopathic effect (CPE) or detecting virus-specific antigen expression. The viral inhibition rate at each concentration was calculated, and the half-maximal effective concentration (EC_50_) was derived by curve fitting.

Calculation of Selectivity Index: The selectivity index (SI), representing the therapeutic window for each compound against the respective virus *in vitro*, was calculated using the formula: SI = CC_50_/EC_50_. All experiments were independently repeated at least three times (Bhattacharya et al., 2021; Salata et al., 2017).

### 4.8 One-step growth curve assay

To evaluate the effect of compound **3b** on the replication kinetics of PEDV, a one-step viral growth curve assay was performed. Briefly, Vero cells were pretreated with a specified concentration of compound **3b** or an equal volume of DMSO (control) for a predetermined duration. Subsequently, the cells were infected with PEDV at the same multiplicity of infection (MOI). Cell culture supernatants were collected at various time points post-infection (0, 6, 12, 24, 36, 44, and 52 h). The viral titers in all samples were determined using the 50% tissue culture infectious dose (TCID_50_) assay on Vero cells and expressed as Log_10_ TCID_50_/mL (Bhattacharya et al., 2021; Salata et al., 2017).

### 4.9 Quantitative PCR (qPCR) detection of viral gene transcription

To analyze the effect of compound **3b** on the transcription of viral genes at different stages, quantitative real-time PCR (qRT-PCR) was performed. Vero cells were pretreated with compound **3b** or DMSO and then infected with PEDV at a consistent multiplicity of infection (MOI). Cells were harvested at 12 and 24 h post-infection, and total RNA was extracted. Subsequently, qRT-PCR was used to specifically detect the mRNA levels of the viral early gene (*IE1*) and the late gene (*VP1*). The relative expression of each gene was calculated using the 2^−ΔΔCt^ method and expressed as fold change relative to the DMSO control group (Ma et al., 2020).

### 4.10 Galectin-3 immunofluorescence staining analysis

TC32 cells or PEDV-infected Vero cells were seeded into confocal dishes and treated with DMSO and compound **3b** for the indicated duration. After treatment, the cells were fixed, permeabilized, and blocked. Subsequently, the cells were incubated with a primary antibody against Galectin-3, followed by incubation with an appropriate fluorescent secondary antibody. Nuclei were counterstained with DAPI. Images were captured randomly under a confocal microscope, and the number of fluorescent puncta per cell was quantified (Fritsch et al., 2016).

### 4.11 Cathepsin B/D activity assay

TC32 cells or PEDV-infected Vero cells subjected to identical treatments were collected and lysed to extract cytosolic proteins. Equal amounts of protein were then incubated with specific fluorescent substrates for Cathepsin B or Cathepsin D under light-protected conditions. The relative enzymatic activity was determined by measuring the fluorescence intensity using a microplate reader at an excitation wavelength of 400 nm and an emission wavelength of 505 nm (Mehanna et al., 2016; Yu et al., 2014).

### 4.12 mRFP-GFP-LC3 autophagic flux analysis

Cells stably expressing the mRFP-GFP-LC3 tandem fluorescent probe were seeded into confocal dishes. They were then treated with DMSO (control), compound **3b**, the autophagy inducer Torin1, or the lysosomal inhibitor chloroquine (CQ). Following treatment, the cells were observed and imaged under a confocal microscope. Yellow puncta (mRFP^+^GFP^+^) represented autophagosomes, while red puncta (mRRP^+^GFP^−^) represented autolysosomes. The puncta were counted, and the average number per cell was calculated for each condition (Bonavita et al., 2025).

### 4.13 Determination of lysosomal lumen pH

Lysosomal pH was measured using the LysoSensor Yellow/Blue staining reagent. Treated cells were incubated with the probe, washed, and then observed under a fluorescence microscope. Quantification was performed using the dual-emission wavelength ratio (Ex/Em: 335/440 nm) characteristic of this probe. The fluorescence ratio was converted to specific pH values based on a standard calibration curve (Ponsford et al., 2021; Xia et al., 2021).

### 4.14 Subcellular fractionation and LC-MS/MS quantification

TC32 cells were treated with 25 μmol/L compound **3b** or DMSO (control) for 6 h. Cells were collected and resuspended in homogenization buffer (0.25 mol/L sucrose, 10 mmol/L HEPES-NaOH, pH 7.2) and disrupted using a Dounce homogenizer. Crude lysosomal fractions were enriched by differential centrifugation, followed by Percoll density gradient centrifugation at 70,000 g for 40 min to purify lysosomes. Fractions were collected, proteins were precipitated with acetonitrile, and compound **3b** concentrations were quantified by LC-MS/MS and normalized to protein content. Three independent replicates were performed.

### 4.15 JC-1 staining for mitochondrial membrane potential

TC32 cells were treated with DMSO, 25 μM compound 3b or 200 μM compound 3b for 6 h. Cells were collected, washed once with PBS, and resuspended in JC-1 working solution prepared according to the manufacturer’s protocol. After incubation at 37 °C for 20 min in the dark, cells were washed twice with cold JC-1 staining buffer and resuspended in 500 μL of staining buffer. Fluorescence was immediately measured using a flow cytometer with 488 nm excitation. Green fluorescence (JC-1 monomer) was detected in the FITC channel (530 nm), and red fluorescence (JC-1 aggregates) was detected in the PE channel (590 nm). The red/green fluorescence ratio was calculated to indicate mitochondrial membrane potential. Data were analyzed from three independent experiments (n = 3).

### 4.16 Statistical analysis

The above results are presented as the mean ± SEM (Standard Error of the Mean) from three independent experiments. Statistical analysis was performed using SPSS 21.0 software, and differences between groups were assessed by one-way analysis of variance (ANOVA).

## Data Availability

The original contributions presented in the study are included in the article/Supplementary Material, further inquiries can be directed to the corresponding author.
