# Supplementary material for: Design of dual-effect molecules based on lysosome-targeting strategy: a mechanistic study on synergistic inhibition of ewing sarcoma and coronaviruses
Source: Front Chem. 2026 Jun 19;14:1851116. doi: 10.3389/fchem.2026.1851116 (PMC13341912; doi:10.3389/fchem.2026.1851116)
Supplement: Supplementary file 1 [file DataSheet1.doc]

**Design of Dual-Effect Molecules Based on Lysosome-Targeting Strategy: A Mechanistic Study on Synergistic Inhibition of Ewing Sarcoma and Coronaviruses**

Shimeng Zhou [[1]](#footnote-2)*, Lu Xu ^2^, Wenlong Ge ^1^

^1^*Cangzhou Integrated Traditional Chinese and Western Medicine Hospital, Hebei Key Laboratory of Integrated Traditional and Western Medicine in Osteoarthrosis Research（Preparing）, Hebei Province, China, 061000*

^2^*Cangzhou People‘s Hospital, Hebei Province, China, 061000*

**Table of Contents**

*^1^H and ^13^C NMR of* **2a**····················································································1

*^1^H and ^13^C NMR of* **2b**····················································································2

*^1^H and ^13^C NMR of* **3a**····················································································3

*^1^H and ^13^C NMR of* **3b**····················································································4

*^1^H and ^13^C NMR of* **3c**····················································································5

*^1^H and ^13^C NMR of* **3d**····················································································6

*HPLC and Mass spectrum of* **3b**······································································7

*Spectral data*·································································································7

*Table S1*········································································································9


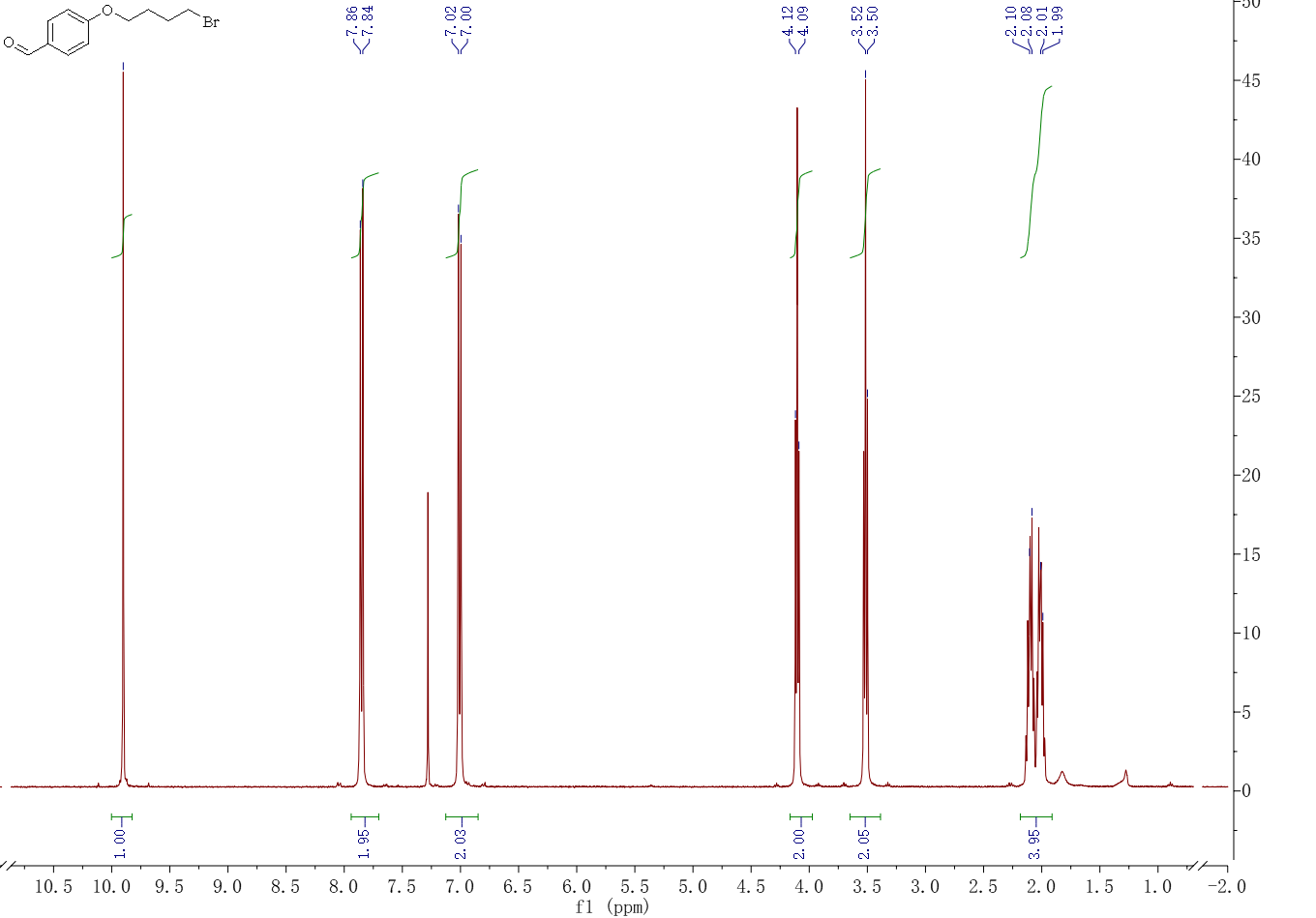


Fig 1. *^1^H NMR of* **2a** (400 MHz, CDCl_3_)


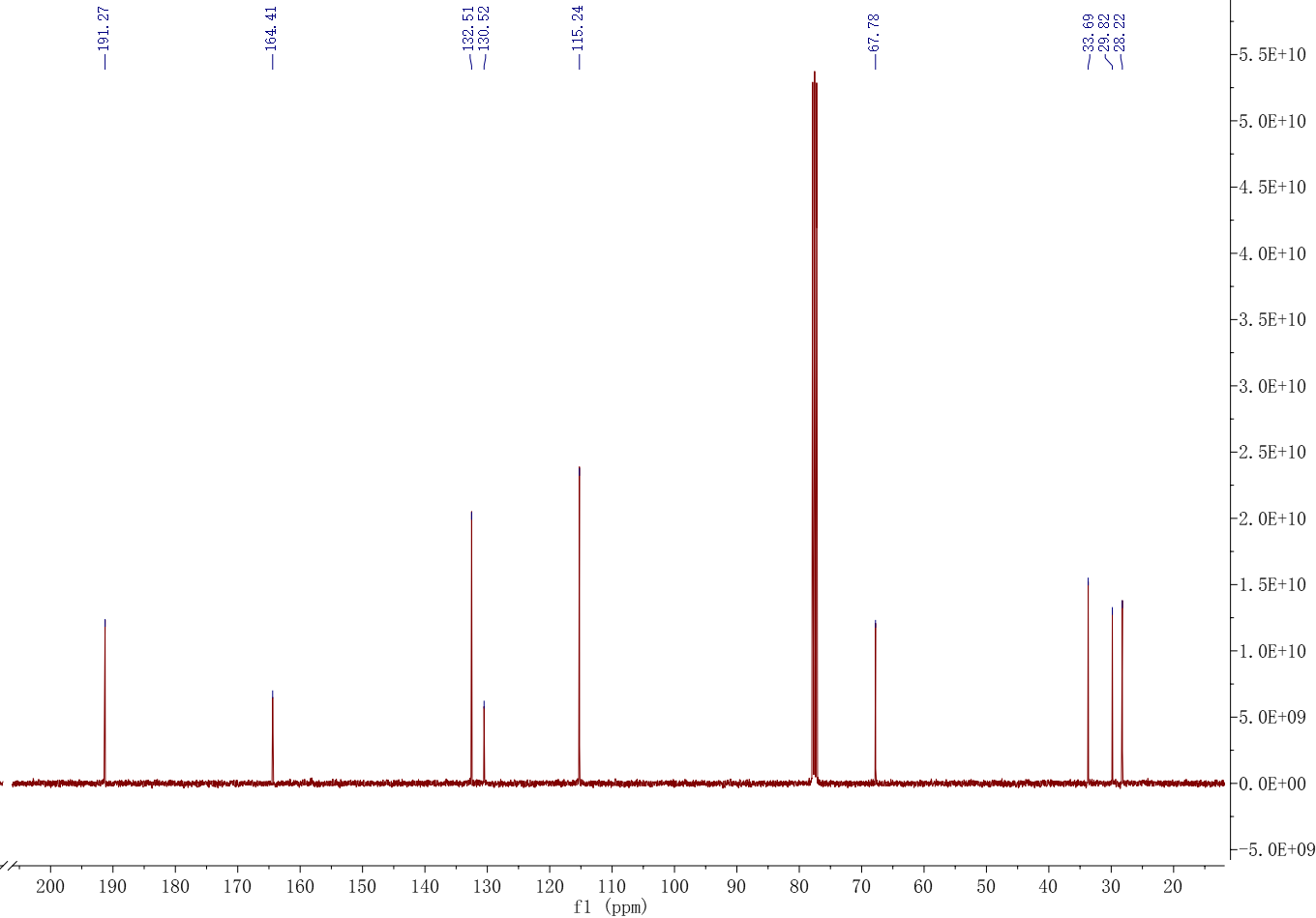


Fig 2. *^13^C NMR of* **2a** (100 MHz, CDCl_3_)


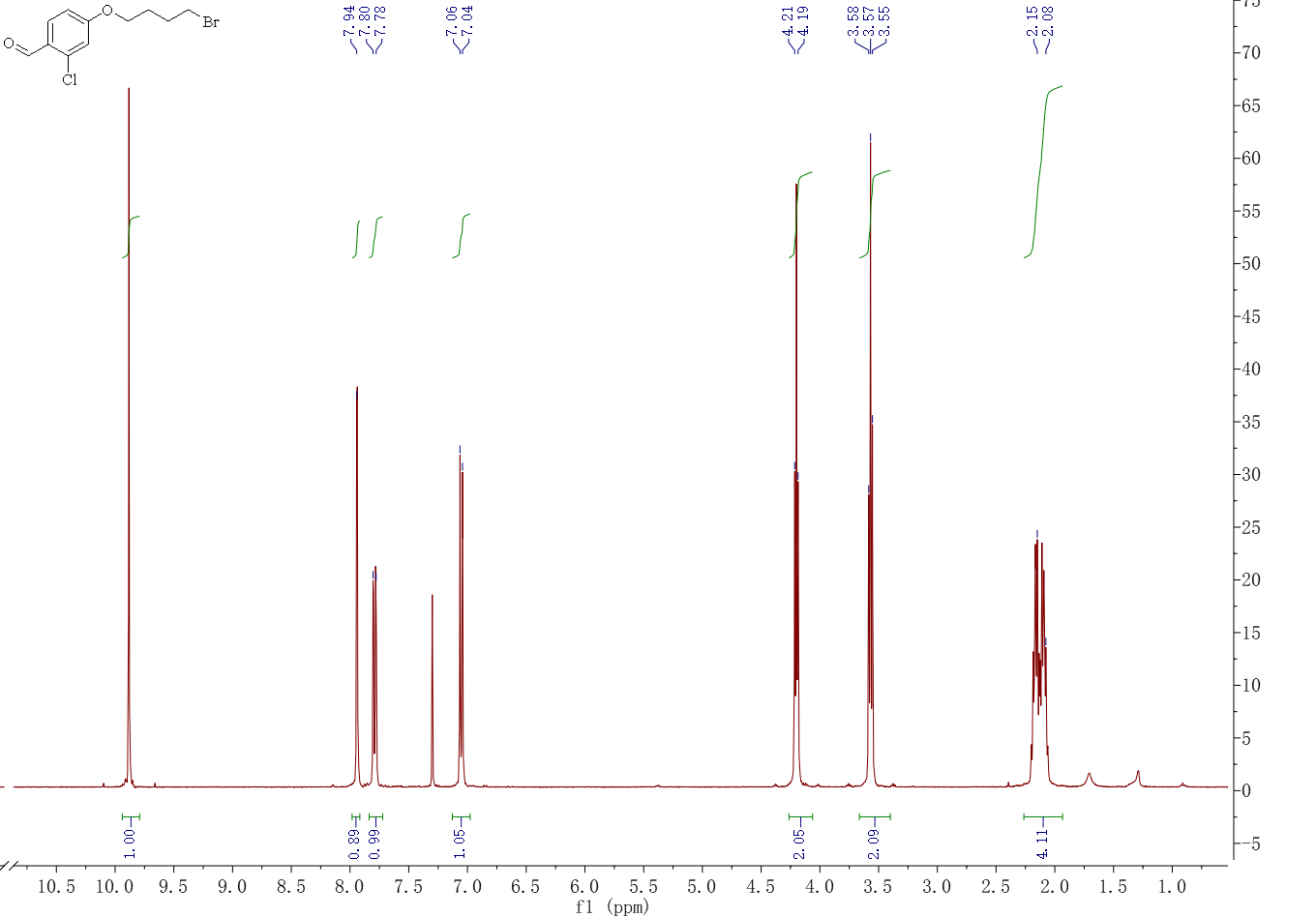


Fig 3. *^1^H NMR of* **2b** (400 MHz, CDCl_3_)


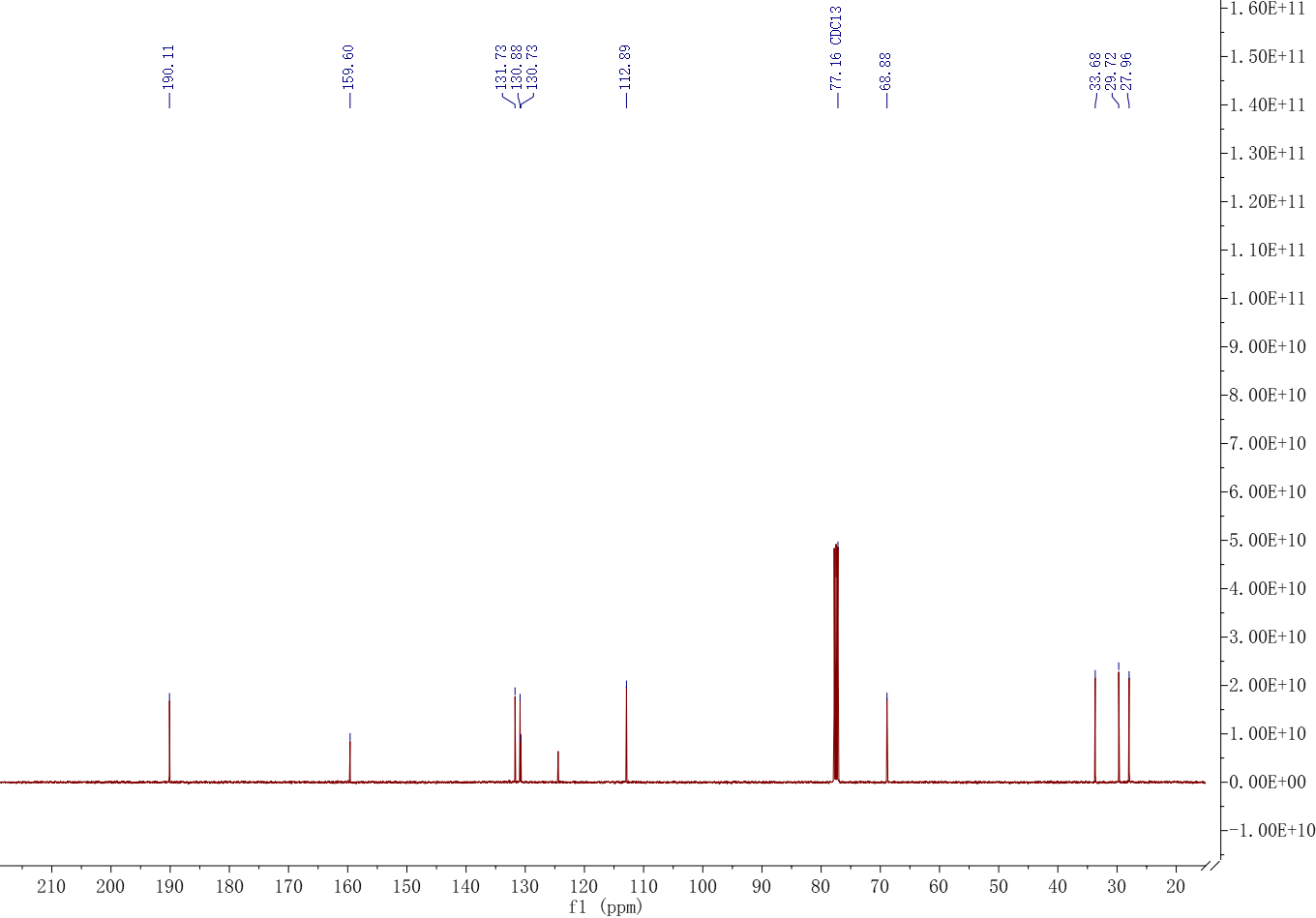


Fig 4. *^13^C NMR of* **2b** (100 MHz, CDCl_3_)


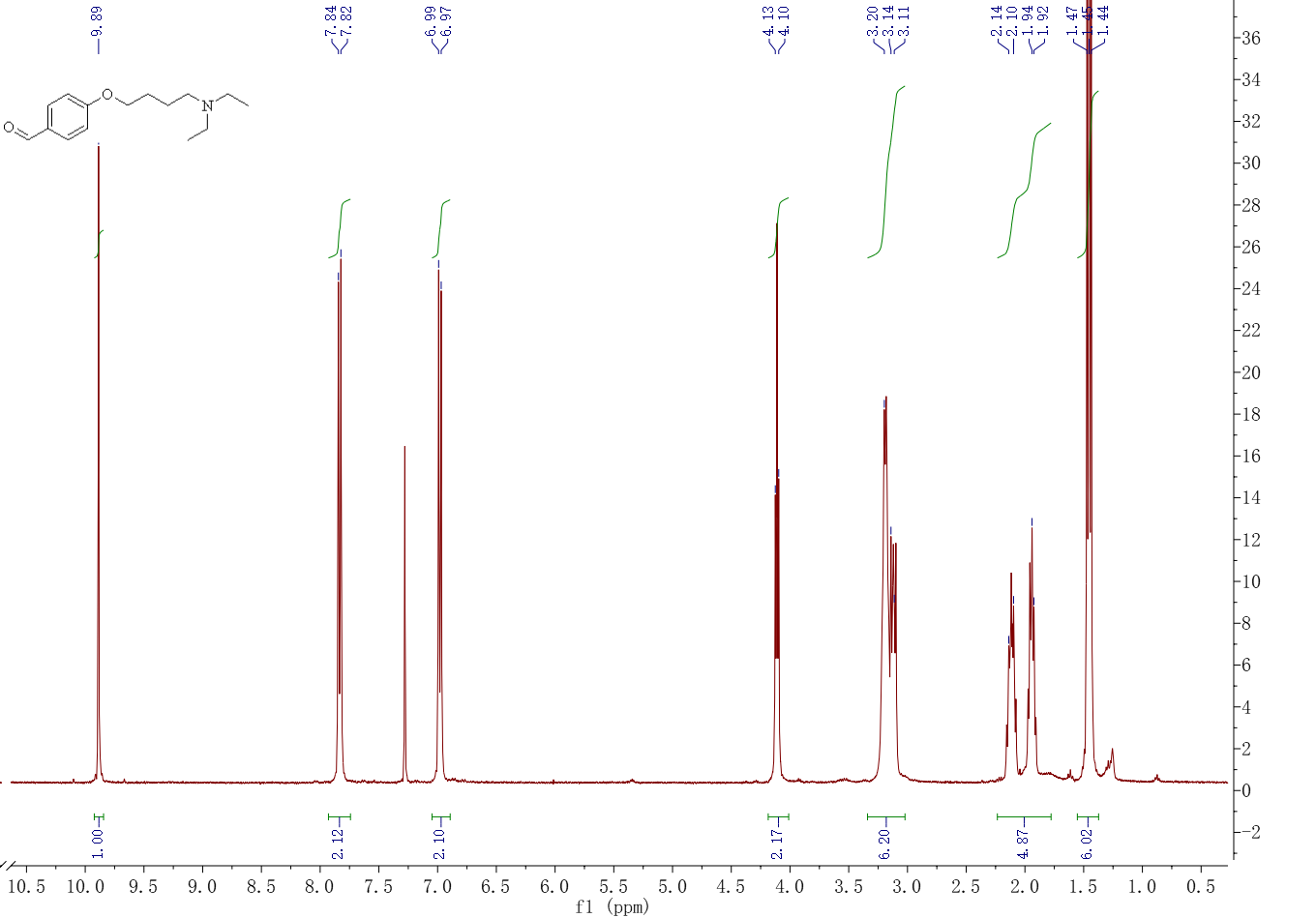


Fig 5. *^1^H NMR of* **3a** (400 MHz, CDCl_3_)


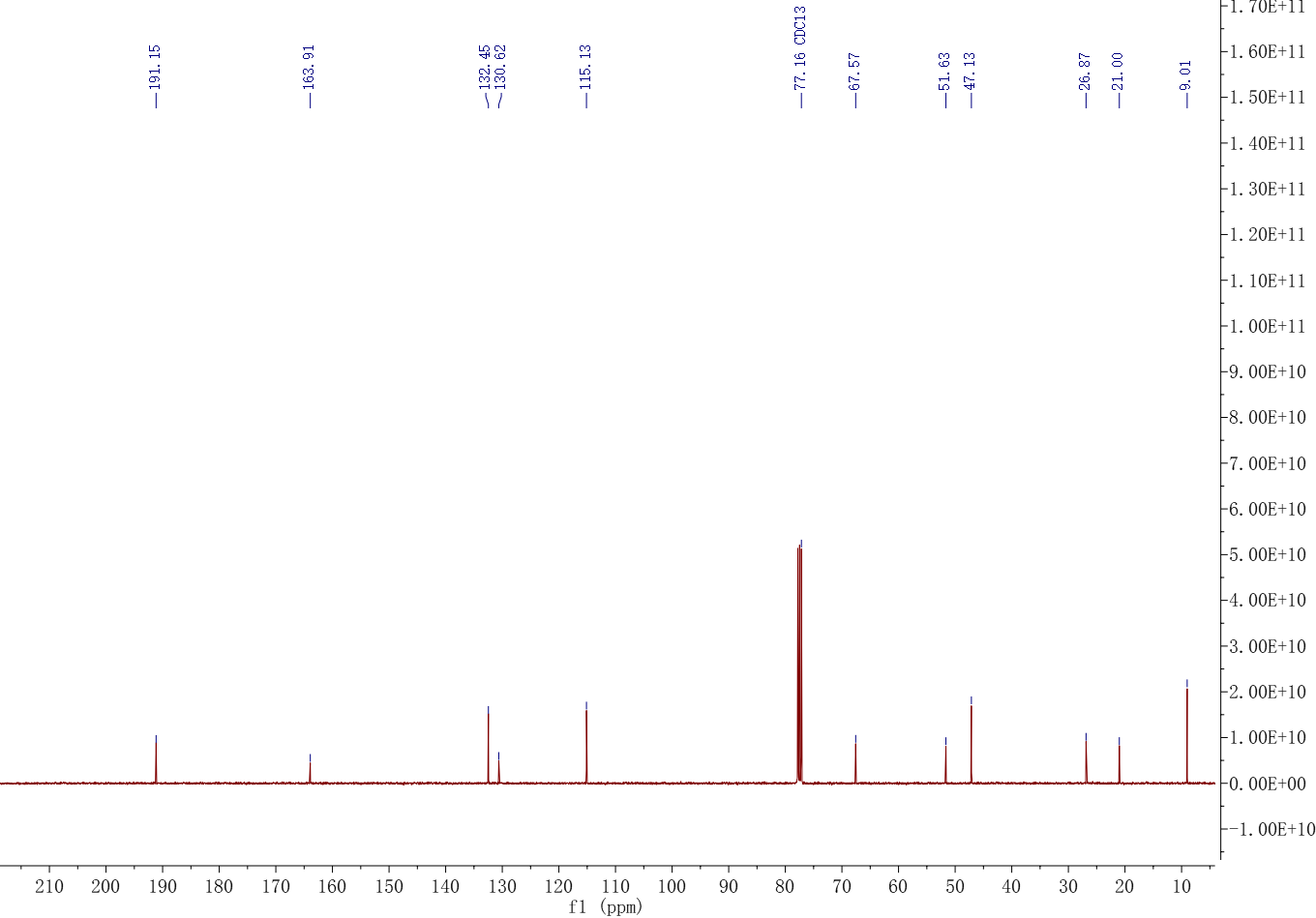


Fig 6. *^13^C NMR of* **3a** (100 MHz, CDCl_3_)


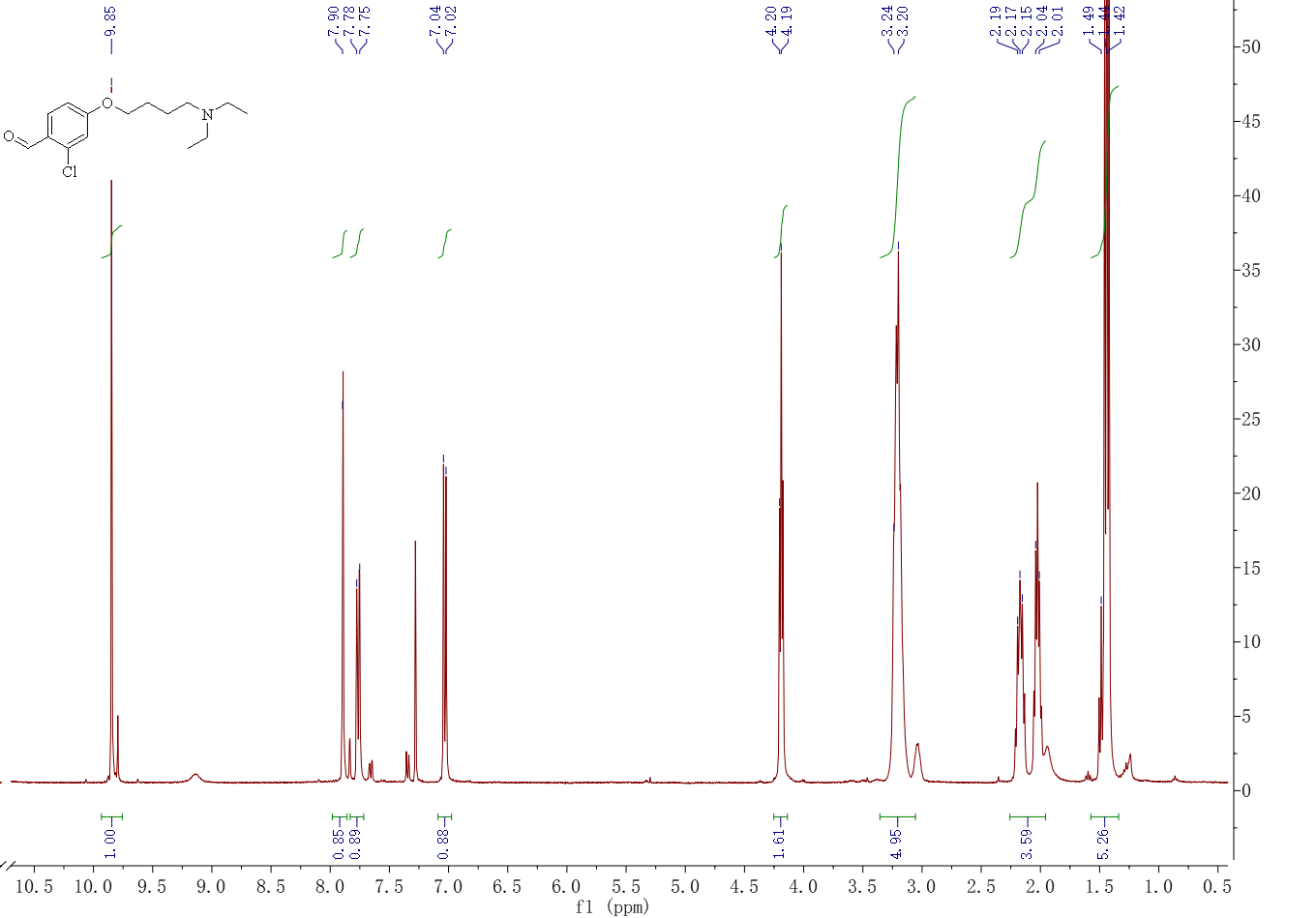


Fig 7. *^1^H NMR of* **3b** (400 MHz, CDCl_3_)


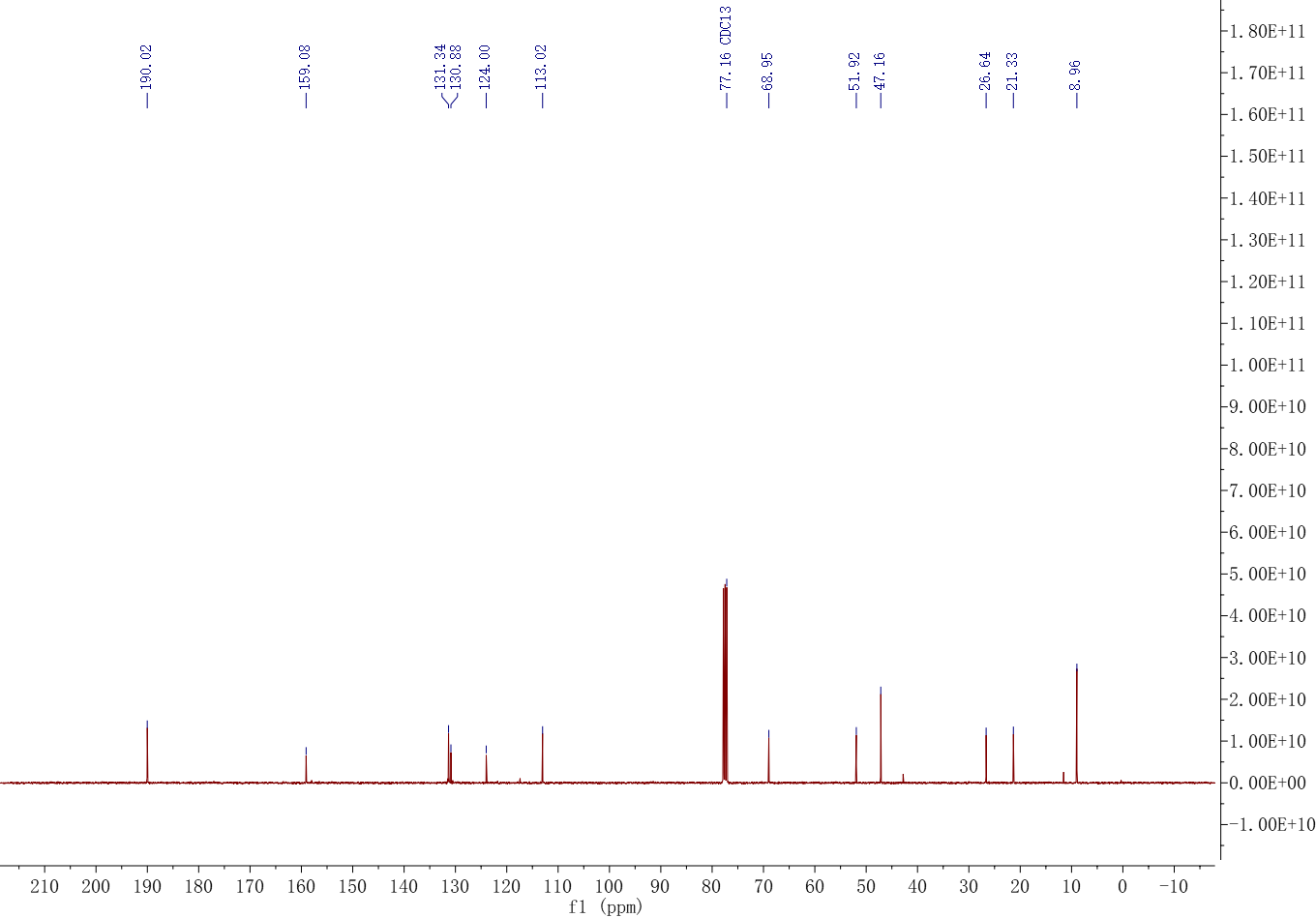


Fig 8. *^13^C NMR of* **3b** (100 MHz, CDCl_3_)


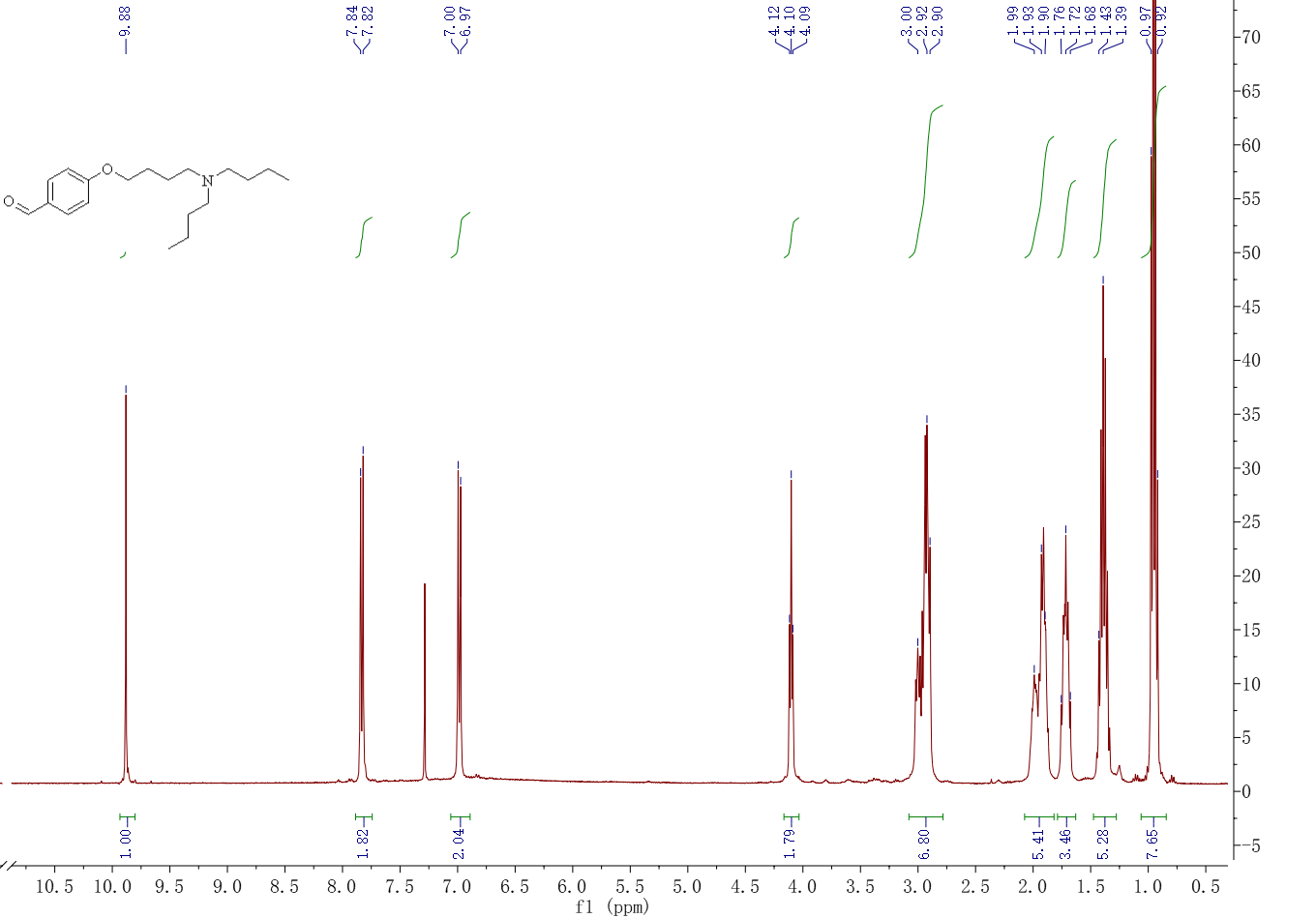


Fig 9. *^1^H NMR of* **3c** (400 MHz, CDCl_3_)


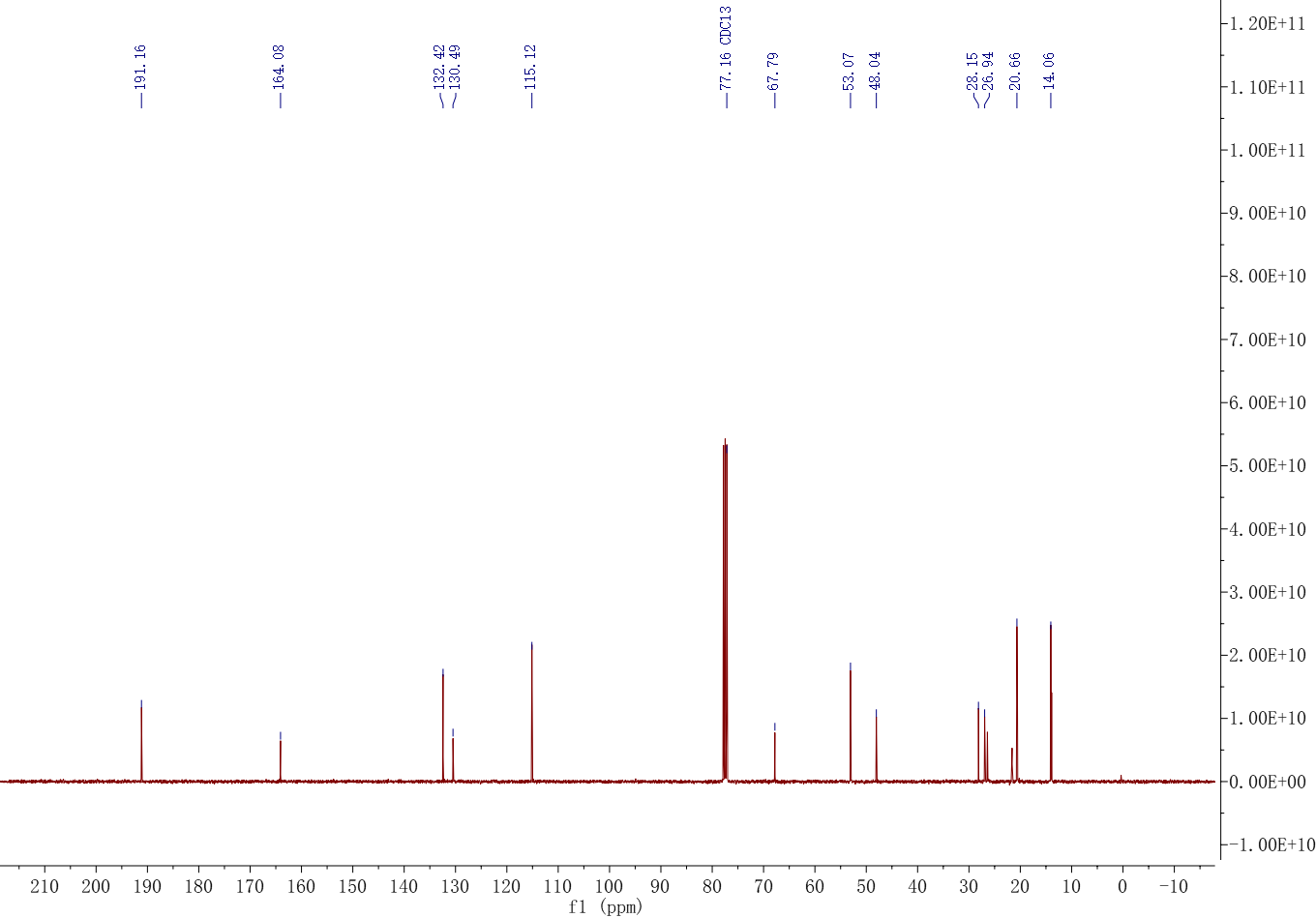


Fig 10. *^13^C NMR of* **3c** (100 MHz, CDCl_3_)


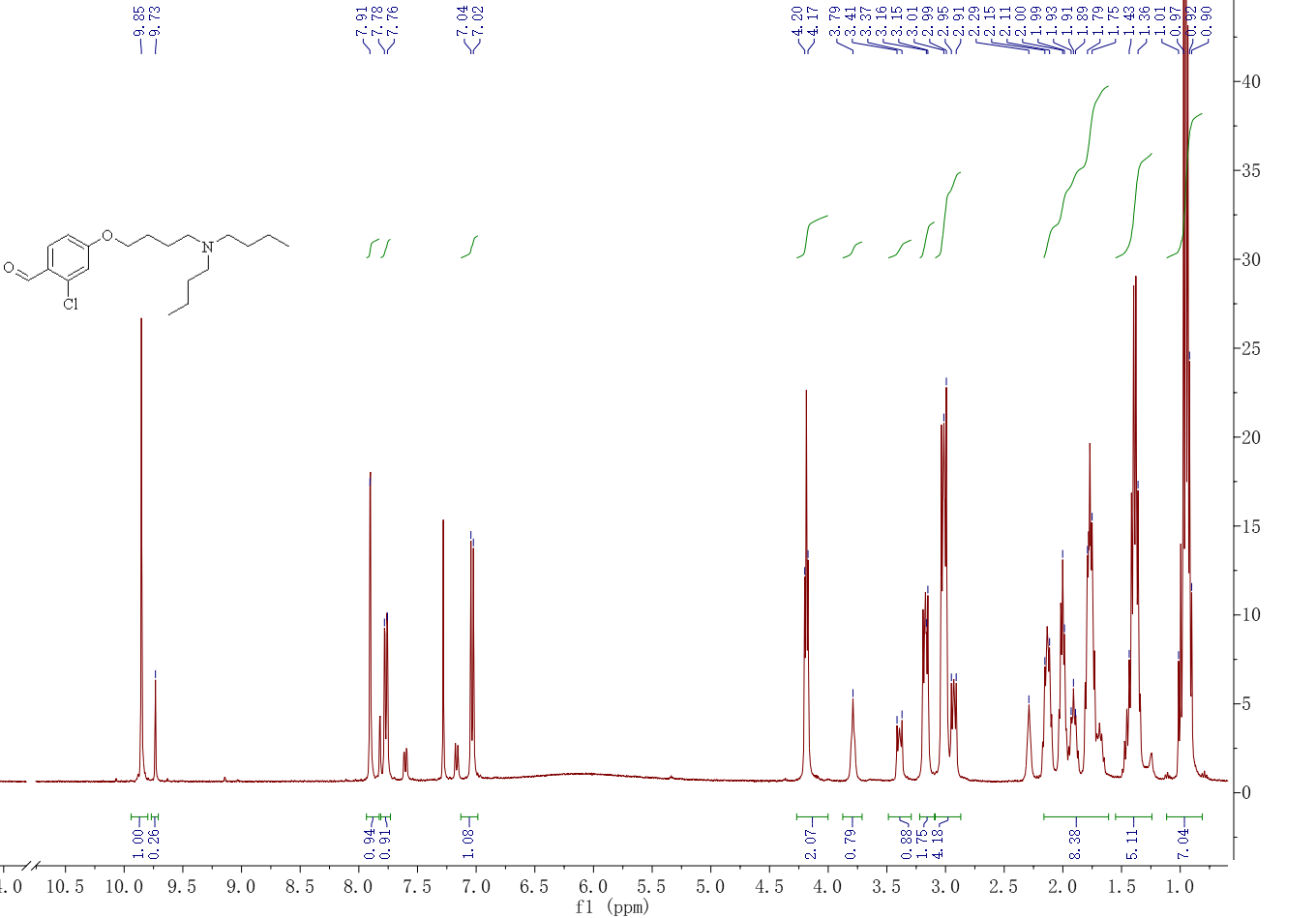


Fig 11. *^1^H NMR of* **3d** (400 MHz, CDCl_3_)


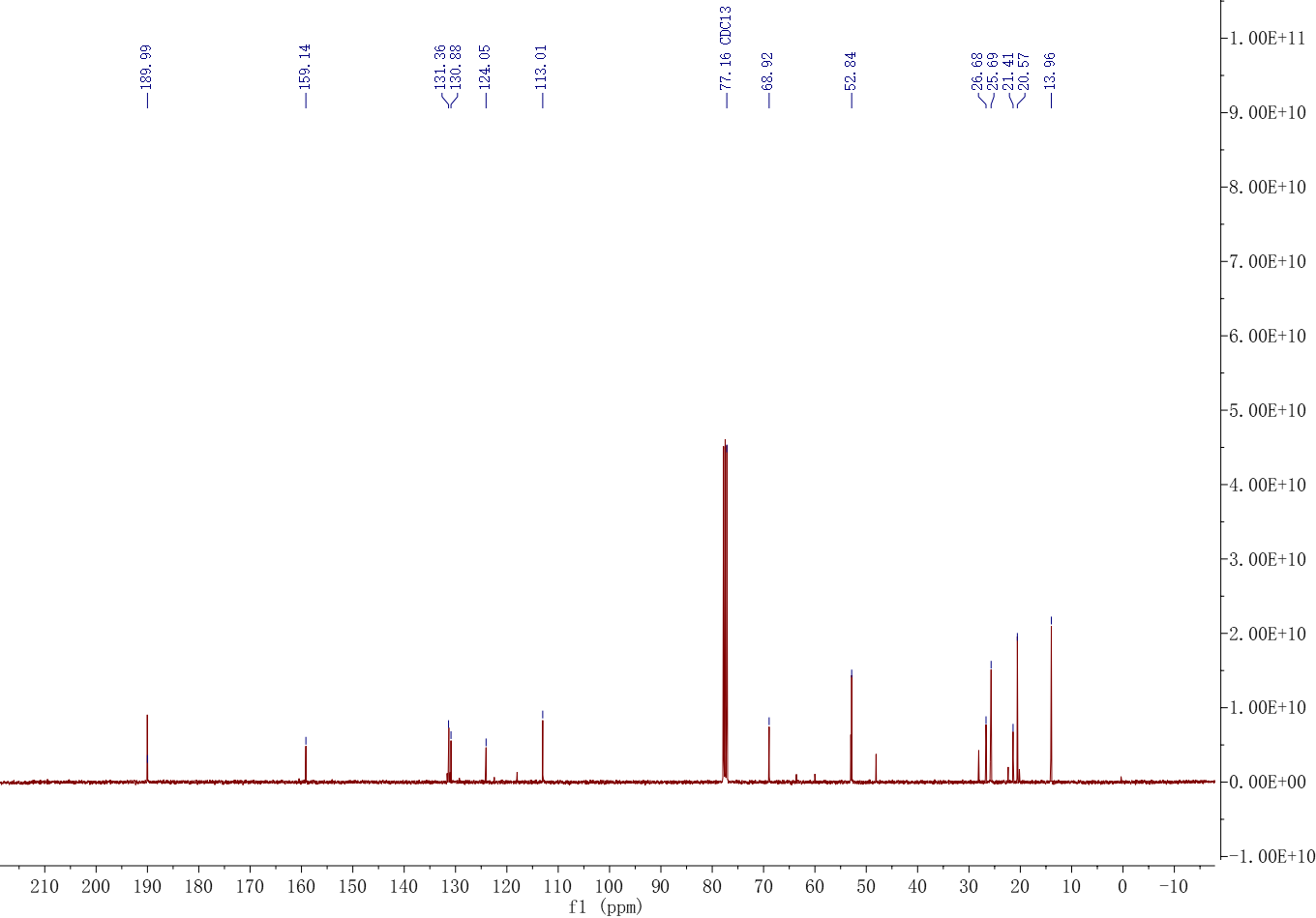


Fig 12. *^13^C NMR of* **3d** (100 MHz, CDCl_3_)


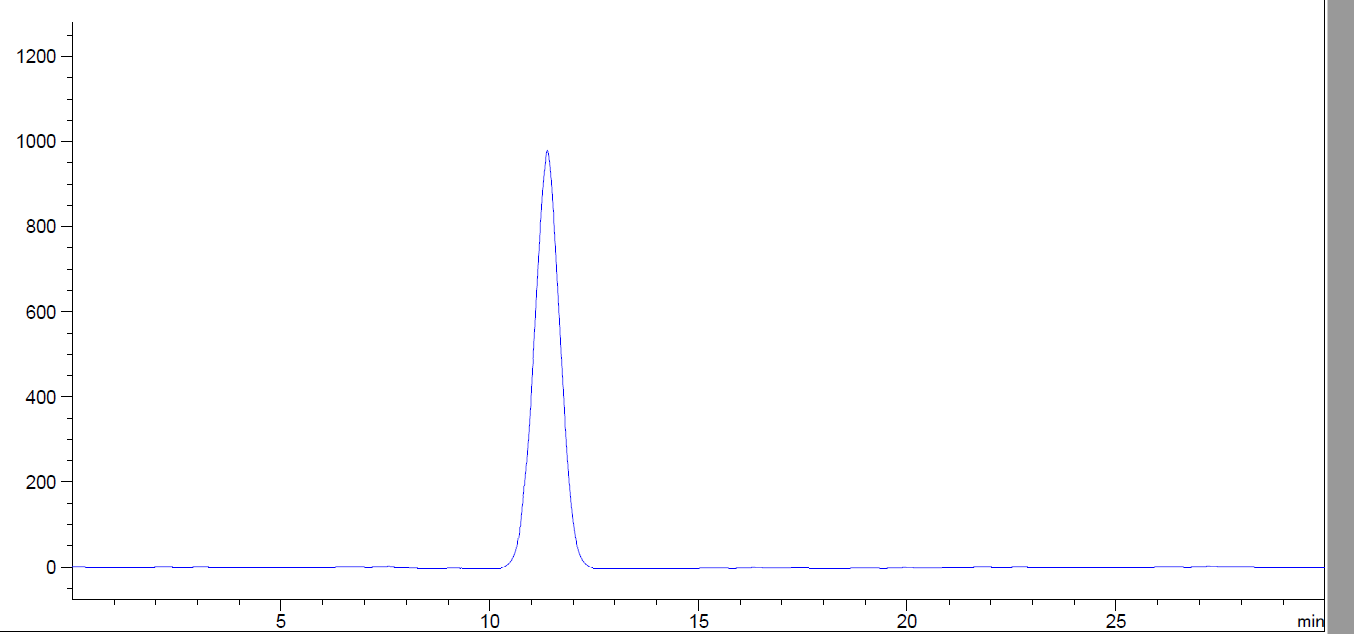


Fig 13. *HPLC spectrum of* **3b**


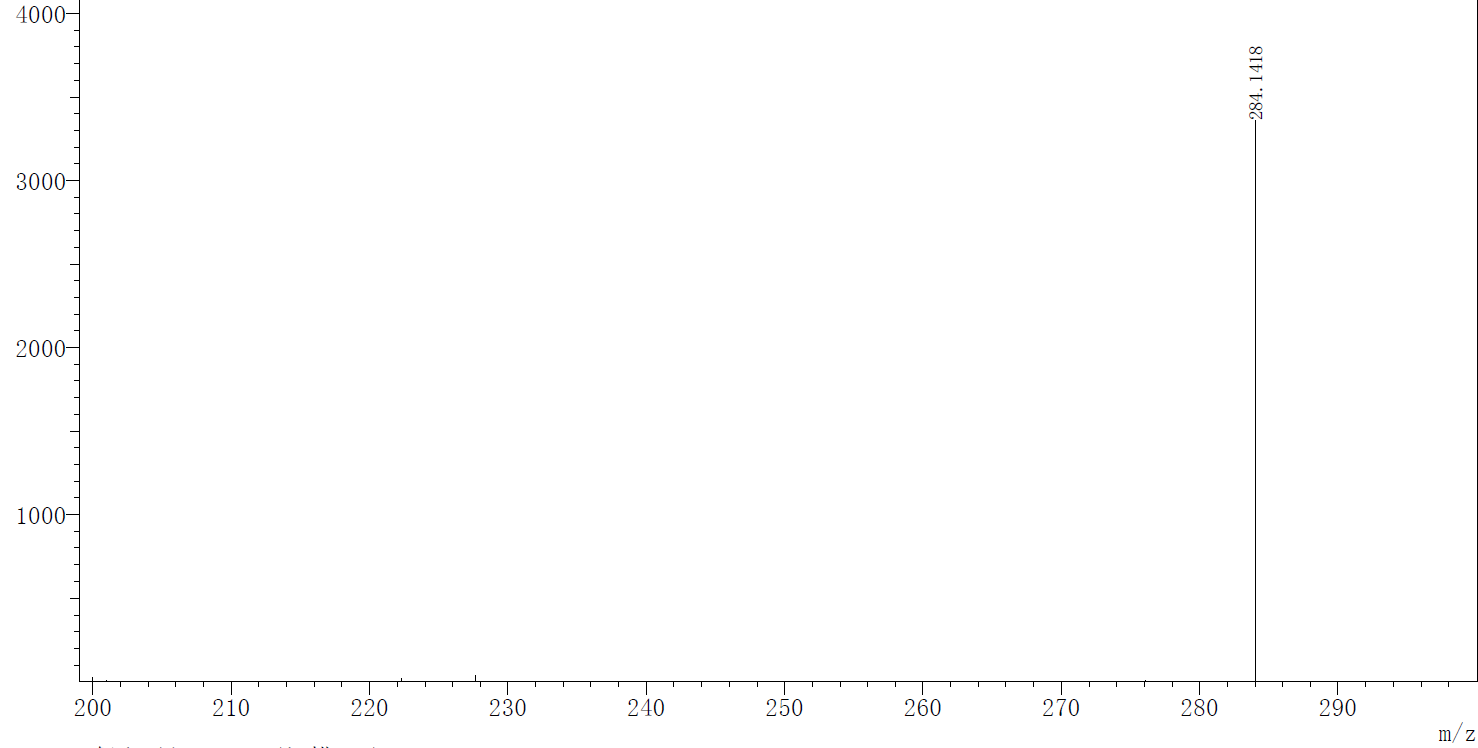


Fig 14. *Mass spectrum of* **3b**

***Spectral data***

4-(4-bromobutoxy)benzaldehyde (**2a**)

**2a** was obtained as a pale yellow solid (179.2 mg, 70% yield). ^1^H NMR (400 MHz, Chloroform-*d*) δ 9.90 (s, 1H), 7.85 (d, *J* = 8.6 Hz, 2H), 7.01 (d, *J* = 8.7 Hz, 2H), 4.10 (d, *J* = 11.8 Hz, 2H), 3.51 (d, *J* = 6.5 Hz, 2H), 2.05 (dd, *J* = 37.8, 7.8 Hz, 4H). ^13^C NMR (101 MHz, Chloroform-*d*) δ 191.27, 132.51, 130.52, 115.24, 67.78, 33.69, 29.82, 28.22. TOF-MS, m/z: [M+H]^+^, calcd. for C_11_H_14_BrO_2_^+^, 257.0177, found: 257.0195.

4-(4-bromobutoxy)-2-chlorobenzaldehyde (**2b**)

**2b** was obtained as a pale yellow solid (237.8 mg, 82% yield). ^1^H NMR (400 MHz, Chloroform-*d*) δ 9.88 (s, 1H), 7.94 (s, 1H), 7.79 (d, *J* = 10.4 Hz, 1H), 7.05 (d, *J* = 8.5 Hz, 1H), 4.20 (d, *J* = 11.6 Hz, 2H), 3.57 (t, *J* = 6.3 Hz, 2H), 2.11 (d, *J* = 29.3 Hz, 4H). ^13^C NMR (101 MHz, Chloroform-*d*) δ 190.11, 159.60, 131.73, 130.88, 130.73, 112.89, 68.88, 33.68, 29.72, 27.96. TOF-MS, m/z: [M+H]^+^, calcd. for C_11_H_13_BrClO_2_^+^, 290.9787, found: 290.9801.

1. (4-(diethylamino)butoxy)benzaldehyde (**3a**)

**3a** was obtained as a pale yellow solid (152.5 mg, 61% yield). ^1^H NMR (400 MHz, Chloroform-*d*) δ 9.89 (s, 1H), 7.83 (d, *J* = 8.6 Hz, 2H), 6.98 (d, *J* = 8.6 Hz, 2H), 4.11 (d, *J* = 11.6 Hz, 2H), 3.34 – 3.02 (m, 6H), 2.23 – 1.78 (m, 5H), 1.45 (t, *J* = 7.3 Hz, 6H). ^13^C NMR (101 MHz, Chloroform-*d*) δ 191.15, 163.91, 132.45, 130.62, 115.13, 67.57, 51.63, 47.13, 26.87, 21.00, 9.01. TOF-MS, m/z: [M+H]^+^, calcd. for C_15_H_24_NO_2_^+^, 250.1807, found: 250.1803.

2-chloro-4-(4-(diethylamino)butoxy)benzaldehyde (**3b**)

**3b** was obtained as a pale yellow solid (213.1 mg, 75% yield). ^1^H NMR (400 MHz, Chloroform-*d*) δ 9.85 (s, 1H), 7.90 (s, 1H), 7.76 (d, *J* = 10.1 Hz, 1H), 7.03 (d, *J* = 8.5 Hz, 1H), 4.20 (d, *J* = 5.6 Hz, 2H), 3.22 (d, *J* = 15.0 Hz, 5H), 2.26 – 1.96 (m, 4H), 1.43 (d, *J* = 7.3 Hz, 5H). ^13^C NMR (101 MHz, Chloroform-*d*) δ 190.02, 159.08, 131.34, 130.88, 124.00, 113.02, 68.95, 51.92, 47.16, 26.64, 21.33, 8.96. TOF-MS, m/z: [M+H]^+^, calcd. for C_15_H_23_ClNO_2_^+^, 284.1417, found: 284.1418.

4-(4-(dibutylamino)butoxy)benzaldehyde (**3c**)

**3c** was obtained as a pale yellow solid (244.1 mg, 80% yield). ^1^H NMR (400 MHz, Chloroform-*d*) δ 9.88 (s, 1H), 7.83 (d, *J* = 8.7 Hz, 2H), 6.98 (d, *J* = 8.7 Hz, 2H), 4.10 (t, *J* = 5.8 Hz, 2H), 3.08 – 2.78 (m, 7H), 2.07 – 1.82 (m, 5H), 1.79 – 1.63 (m, 3H), 1.41 (d, *J* = 14.8 Hz, 5H), 0.95 (d, *J* = 22.3 Hz, 8H). ^13^C NMR (101 MHz, Chloroform-*d*) δ 191.16, 164.08, 132.42, 130.49, 115.12, 67.79, 53.07, 48.04, 28.15, 26.94, 20.66, 14.06. TOF-MS, m/z: [M+H]^+^, calcd. for C_19_H_32_NO_2_^+^, 306.2433, found: 306.2445.

2-chloro-4-(4-(dibutylamino)butoxy)benzaldehyde (**3d**)

**3d** was obtained as a pale yellow solid (244.1 mg, 72% yield). ^1^H NMR (400 MHz, Chloroform-*d*) δ 9.85 (s, 1H), 9.73 (s, 0H), 7.91 (s, 1H), 7.77 (d, *J* = 10.3 Hz, 1H), 7.03 (d, *J* = 8.5 Hz, 1H), 4.19 (d, *J* = 11.2 Hz, 2H), 3.79 (s, 1H), 3.39 (d, *J* = 16.9 Hz, 1H), 3.16 (d, *J* = 5.0 Hz, 2H), 3.09 – 2.87 (m, 4H), 2.16 – 1.61 (m, 8H), 1.40 (d, *J* = 30.1 Hz, 5H), 1.12 – 0.81 (m, 7H). ^13^C NMR (101 MHz, Chloroform-*d*) δ 189.99, 159.14, 131.36, 124.05, 113.01, 68.92, 52.84, 26.68, 25.69, 21.41, 20.57, 13.96. TOF-MS, m/z: [M+H]^+^, calcd. for C_19_H_31_ClNO_2_^+^, 340.9118, found: 340.9124.

**Table S1**. Distribution of TC32 cell subpopulations after Annexin V/PI staining.

| **Cell population** | **Control (%)** | **Compound 3b (200 μM) (%)** |
| --- | --- | --- |
| Live | 93.2 | 61.5 |
| Early apoptotic | 2.1 | 18.3 |
| Late apoptotic | 1.3 | 14.7 |
| Necrotic | 3.4 | 5.5 |

1. * Correspondent. E-mail: m19331789399@163.com [↑](#footnote-ref-2)
